# Garlic oil alleviates high triglyceride levels in alcohol‐exposed rats by inhibiting liver oxidative stress and regulating the intestinal barrier and intestinal flora

**DOI:** 10.1002/fsn3.2854

**Published:** 2022-03-29

**Authors:** Yanhui Wang, Huaqi Zhang, Xiangyun Teng, Peiyu Guo, Yuwei Zuo, Hui Zhao, Peng Wang, Hui Liang

**Affiliations:** ^1^ Department of Nutrition and Food Hygiene School of Public Health Qingdao University Qingdao China

**Keywords:** garlic oil, intestinal flora, lipid metabolism, oxidative stress

## Abstract

Garlic oil (GO) is a kind of natural extract extracted from garlic, which has strong antioxidant activity. This study elucidates the protective mechanism of GO against alcohol‐induced high triglyceride levels. Sixty male Sprague Dawley rats were assigned to five groups, including a control group (CON), a model group (MOD) treated with alcohol 56% v/v at 8 ml kg^−1^ day^−1^ for 2 weeks then 10 ml kg^−1^ day^−1^ for 8 weeks, a low‐dose GO group (GO‐L) given GO at 20 mg kg^−1^ day^−1^, a high‐dose GO group (GO‐H) given GO at 40 mg kg^−1^ day^−1^, and a positive group (POS) given diammonium glycyrrhizinate at 200 mg kg^−1^ day^−1^. The results showed that GO could significantly reduce the serum and liver triglyceride levels caused by alcohol exposure (*p* < .05). The GO‐H group significantly reduced MDA level, increased SOD and GSH‐Px levels in serum, liver, and colon (*p* < .05), significantly increased the levels of Sirt1 and PGC‐1α proteins and reduced FoxO1 protein level in liver (*p* < .05), and significantly increased the levels of ZO‐1 and Claudin1 proteins in the colon compared to the MOD group (*p* < .05). The 16S rRNA sequencing showed that the intestinal flora of the GO‐H group was significantly changed compared with the MOD group. In summary, GO has the potential to improve high triglyceride levels in serum and liver induced by alcohol exposure, which may be related to the inhibition of oxidative stress regulation of Sirt1 and its downstream proteins, and to the restoration of the intestinal barrier and intestinal flora.

## Graphical abstract

GO can alleviate the high triglyceride levels caused by alcohol exposure by regulating Sirt1 and its downstream proteins FoxO1 and PGC‐1α to regulate oxidative stress. GO can also repair the mechanical barrier of intestinal mucosa and regulate intestinal flora, which may be related to the reduction of high triglyceride levels in alcohol‐exposed rats.

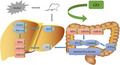

## 1 INTRODUCTION

Alcohol intake can lead to lipid metabolism disorders in the body (Liu et al., 2018), of which abnormal triglyceride (TG) accumulation is one of the most common. Significant evidence shows that the pathogenesis of lipid metabolism disorder is related to increased oxidative stress (Albano, 2006; Dey & Cederbaum, 2006), which can cause the concentration of free radicals to exceed a critical level, disrupting homeostasis. The products of oxidative stress accumulate in the body causing cytotoxicity, damaging liver cells, and causing lipid metabolism disorders (Wang et al., 2019).

In humans, large amounts of alcohol can be metabolized to acetaldehyde and acetic acid under the catalysis of ethanol dehydrogenase and acetaldehyde dehydrogenase (Beier et al., 2011). The increase of acetaldehyde caused by ethanol can destroy mitochondria and inhibit the tricarboxylic acid cycle. Alcohol metabolism also produces excess free radicals which consume a lot of antioxidants, resulting in an imbalance of oxidation and antioxidant effects in the body, accumulation of lipid peroxides, and liver cell damage (Leung & Nieto, 2013; Tang et al., 2014). Silent information regulator 1 (Sirt1) is a NAD^+^‐dependent protein deacetylase, which plays an important role in cell differentiation, aging, apoptosis, transcriptional regulation, signal transduction, oxidative stress, and other important biological processes. Studies have shown that Sirt1 can regulate forkhead transcription factor O1 (FoxO1) expression through deacetylation and that the acetylation level of FoxO1 is related to oxidative stress, affecting the expression of TG transporters and lipid metabolism (Carlomosti et al., 2017; Zhang, Gui, et al., 2019). Many studies have confirmed that Sirt1 can activate the expression level of proliferator‐activated receptor‐gamma coactivator‐1alpha (PGC‐1α), and that PGC‐1α can activate the activity of oxidase, increasing β‐oxidation and reducing TG production (Aguirre‐Rueda et al., 2015; Waldman et al., 2018).

Alcohol direct stimulation can affect gastrointestinal function and cause changes in the intestinal barrier function by excessive production of oxygen free radicals in intestinal tissues and lumens, resulting in enhanced permeability of intestinal epithelial cells (Muccioli et al., 2010). The long‐term stimulation of oxidative stress destroys the environment in which the bacteria live and when oxidative stress occurs, intestinal epithelium passively diffuses oxidation products, increasing the oxidation potential, stimulating the growth of aerobic bacteria, and then changing the composition of intestinal flora (Reese et al., 2018). It has been reported that free radicals can directly attack intestinal microorganisms because superoxide anion may penetrate the biofilm through anion channels (Chance et al., 1979). In recent years, many studies have shown that changes in intestinal flora play an important role in the occurrence and development of many diseases (Gart et al., 2018; Schroeder & Bäckhed, 2016). One study showed that exposure to alcohol caused an overgrowth of pathogenic bacteria and a decrease in beneficial intestinal bacteria (Cresci et al., 2017). Disturbances in intestinal microbial composition can disrupt the intestinal barrier function and liver lipid metabolism through a variety of pathways (Guo et al., 2018). Bacterial metabolites such as short‐chain fatty acids (SCFAs) regulate lipid metabolism by increasing energy consumption and reducing TG accumulation in the liver (Schoeler & Caesar, 2019).

Garlic oil (GO) is a commercial garlic product prepared by steam distillation, which has been found to have a variety of pharmacological effects, which are related to its active compounds (Balaha et al., 2016; El‐Akabawy & El‐Sherif, 2016), mainly diallyl trisulfide (DATS) and diallyl disulfide (DADS) (Agarwal, 1996; Nicastro et al., 2015). A range of biological uses of GO have been reported, including antiatherosclerosis, antihypertension, antibacterial, anticancer, and immunomodulatory (Agarwal, 1996), and these functions are mainly attributed to its antioxidant activity (Banerjee et al., 2003). There have been few studies on whether GO can regulate lipid metabolism disorders in alcohol‐exposed rats by regulating oxidative stress, the intestinal barrier, and the intestinal flora.

In this study, rats were given alcohol and GO by gavage for 10 weeks to explore the regulatory effect of GO on lipid metabolism disorders in alcohol‐exposed rats and to explore the possible role of GO in regulating oxidative stress, intestinal barrier, and intestinal flora.

## 2 MATERIALS AND METHODS

### 2.1 Chemicals and reagents

The GO was purchased from Anhui Kaibo Biotechnology Co., Ltd. The top seven organosulfur compounds with the highest content were diallyl trisulfide (DATS) (45.00%), diallyl disulfide (DADS) (26.62%), diallyl sulfide (8.20%), methyl allyl trisulfide (7.37%), diallyl tetrathioether (3.17%), methyl allyl disulfide (2.77%), and methyl allyl monothioether (1.45%). DATS and DADS are the main organosulfur compounds.

### 2.2 Animals and experimental design

A total of 60 six‐week‐old male Sprague Dawley rats weighing 180–220 g were obtained from Shandong Lukang Pharmaceutical Co., Ltd. (license no.: SCXK 2014–0007). These rats were maintained in an environmentally controlled animal house with a temperature of 23 ± 2°C and humidity of 50 ± 10% with a 12‐h light/dark cycle. All animals had free access to balanced rat chow and water and were given an adaptive feeding regimen for 1 week before the experiment.

**FIGURE 1:**
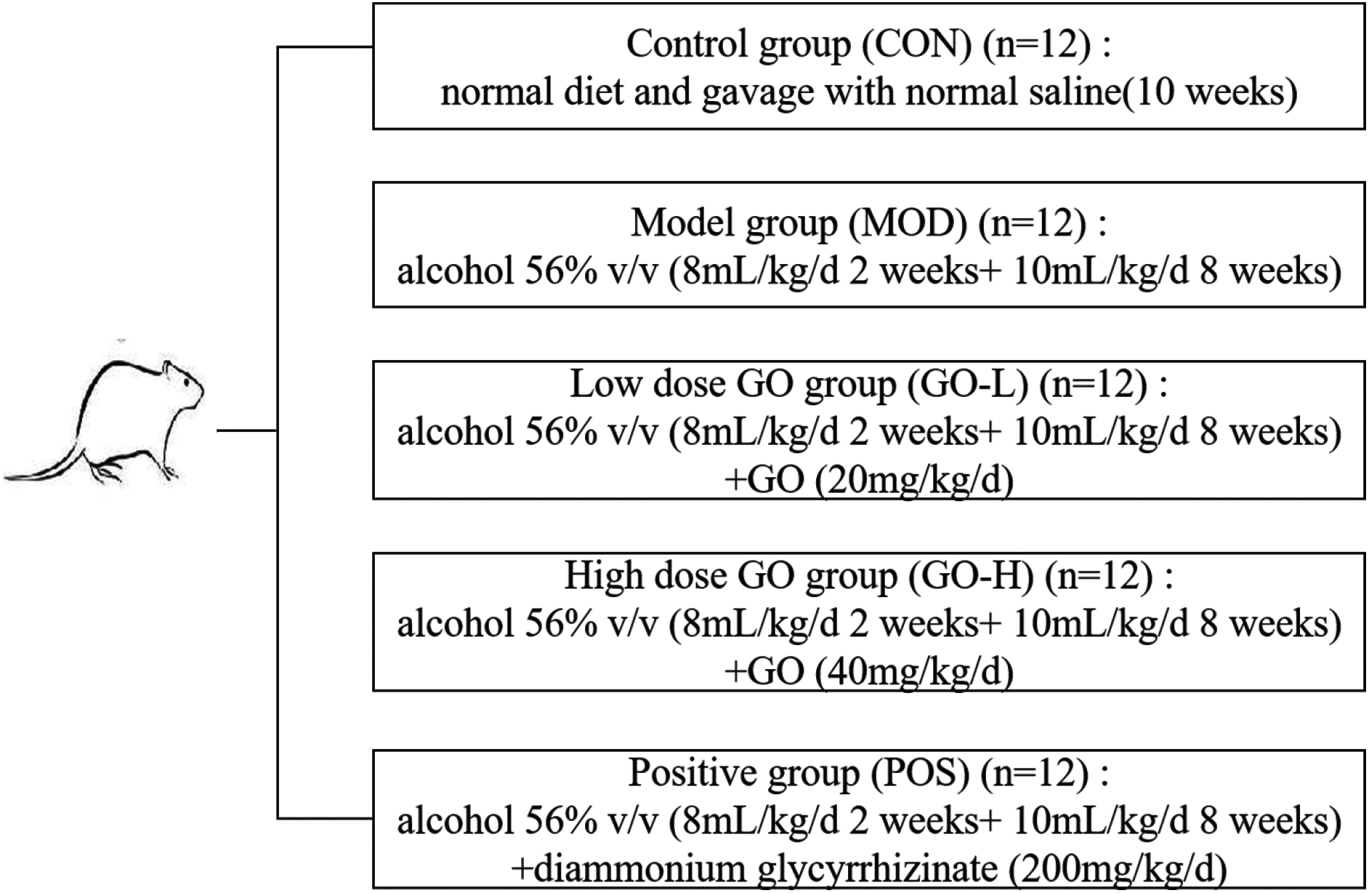
Animal grouping and intervention

After adaptive feeding, the animals were randomly assigned to five groups of 12 animals based on body weight (Figure 1). The normal diets (AIN‐93M) were purchased from Shandong Lukang Pharmaceutical Co., Ltd. The type of alcohol was Red Star Erguotou 56% (v/v) alcohol purchased from Beijing Red Star Co., Ltd.

After the last treatment, all rats were fasted overnight for 12 h, and then euthanized with pentobarbital. Blood was collected from the abdominal aorta and the liver and intestines were surgically removed. After the blood rested for 10–20 min, it was centrifuged at 1006 *g* for 20 min and the serum was stored at −80°C until analyzed. The liver was weighed after hepatectomy, then the liver and intestines were immediately placed on liquid nitrogen, and then stored at −80°C for further analysis.

All animals were fed and operated with the approval of the Animal Care and Use Committee of the Medical College of Qingdao University, following the institution's guidelines for the care and use of experimental animals (approval number: no. 20190828SD601106002).

### 2.3 Biochemical analysis of blood samples

According to our previous method (Liu et al., 2021), the serum levels of alanine aminotransferase (ALT), glutamic oxalacetic transaminase (AST), gamma‐glutamyl transferase (GGT), cholinesterase (CHE), TG, total cholesterol (TC), high‐density lipoprotein cholesterol (HDL‐C), and low‐density lipoprotein cholesterol (LDL‐C) were measured with an automated analyzer for clinical chemistry (Nanjing Jiancheng Bioengineering Institute, Nanjing, China). Superoxide dismutase (SOD), glutathione peroxidase (GSH‐Px), and malondialdehyde (MDA) levels in the supernatant were measured with determination kits (Nanjing Jiancheng Bioengineering Institute).

### 2.4 Detection of TG level in liver tissue

Liver homogenate was prepared according to the previous method (Yan et al., 2022). The TG level in the supernatant was measured with a determination kit (Nanjing Jiancheng Bioengineering Institute).

### 2.5 Detection of oxidative stress index in liver and colon tissues

The levels of SOD, GSH‐Px, and MDA levels in liver and colon tissues were measured with determination kits (Nanjing Jiancheng Bioengineering Institute) according to the manufacturer's instructions.

### 2.6 Western blotting

Liver and colon tissue were resected, homogenized, and centrifuged (15,000 rpm for 5 min). The protein supernatants were collected and then total protein was extracted with tissue protein extraction reagent (T‐PER, Pierce Biotechnology). Total protein content was determined using the BCA assay kit according to the manufacturer's protocol. The proteins in each sample were separated by SDS‐PAGE (10%–12% gel) and then transferred to a PVDF membrane (Millipore Corp.). PVDF membrane was sealed with 5% skimmed milk at room temperature for 1 h and incubated with primary antibody at 4°C overnight. After washing with Tris‐buffered saline (TBS) for three times, the membranes and corresponding secondary antibodies were incubated at room temperature for 1 h. The materials used included anti‐ZO‐1 (Sangon) and anti‐Claudin1, anti‐Sirt1, anti‐PGC‐1α, anti‐FoxO1, and corresponding secondary antibody (Santa Cruz Biotechnology).

### 2.7 16S rRNA gene sequencing

Intestinal contents of rats in the CON, MOD, and GO‐H groups were characterized by 16S rRNA gene sequencing. The method was based on our previous research (Jiang et al.,  2020). The total DNA was examined by Thermo Nanodrop 2000 UV microspectrophotometer and 1% agarose gel electrophoresis. The V3–V4 regions of the 16S rRNA gene were amplified by polymerase chain reaction (PCR). The primers used were 341F: 5′‐CCTACGGGRSGCAGCAG‐3’ and 806R: 5′‐GGACTACVVGGGTATCTAATCAT‐3′. Using the diluted genomic DNA as the template, PCR was performed using the Kapa Hifi Hotstart ReadyMix PCR kit high‐fidelity enzyme. PCR products were detected by 2% agarose gel electrophoresis, and the PCR products were recovered by gel cutting with AxyPrep DNA gel recovery kit (AxyGen Corp.). After recovery, the library was tested by Thermo Nanodrop 2000 UV microspectrophotometer and 2% agarose gel electrophoresis. After the library quality is qualified, Qubit is used for library quantification, and corresponding proportion of mixing is carried out according to the data volume requirements of each sample. Illumina Novaseq PE250 was used for computer sequencing. 16S specific primers were designed to amplify specific regions, and a 425 bp amplified fragment was obtained. In addition, Illumina platform was used to sequence PE250 paired‐end data, and the long sequence was spliced to perform 16S analysis.

### 2.8 Detection of short‐chain fatty acids (SCFAs) in feces

The method was based on our previously published article (Xue, Liang, et al., 2020). Feces (0.5 g) was taken, 500 μl of distilled water was added for homogenizing, and the mixture was left for 20 min and shaken for 3 min. The homogenize was centrifuged at 7155 *g* for 8 min and supernatant was collected, then the supernatant was centrifuged at 7155 *g* for 3 min. The supernatant was filtered and used for the analysis of SCFAs in feces by gas chromatography–mass spectrometry (GC‐MS).

### 2.9 Data analysis

The statistical software SPSS version 22.0 was used for analysis. All quantitative data are presented as the mean ± standard deviation (*SD*). Significance was determined by one‐way analysis of variance followed by LSD test. The Kruskal–Wallis test was used to analyze nonparametric data. The *p* < .05 were considered statistically significant.

## 3 RESULTS

### 3.1 Effects of GO on body weight

**FIGURE 2:**
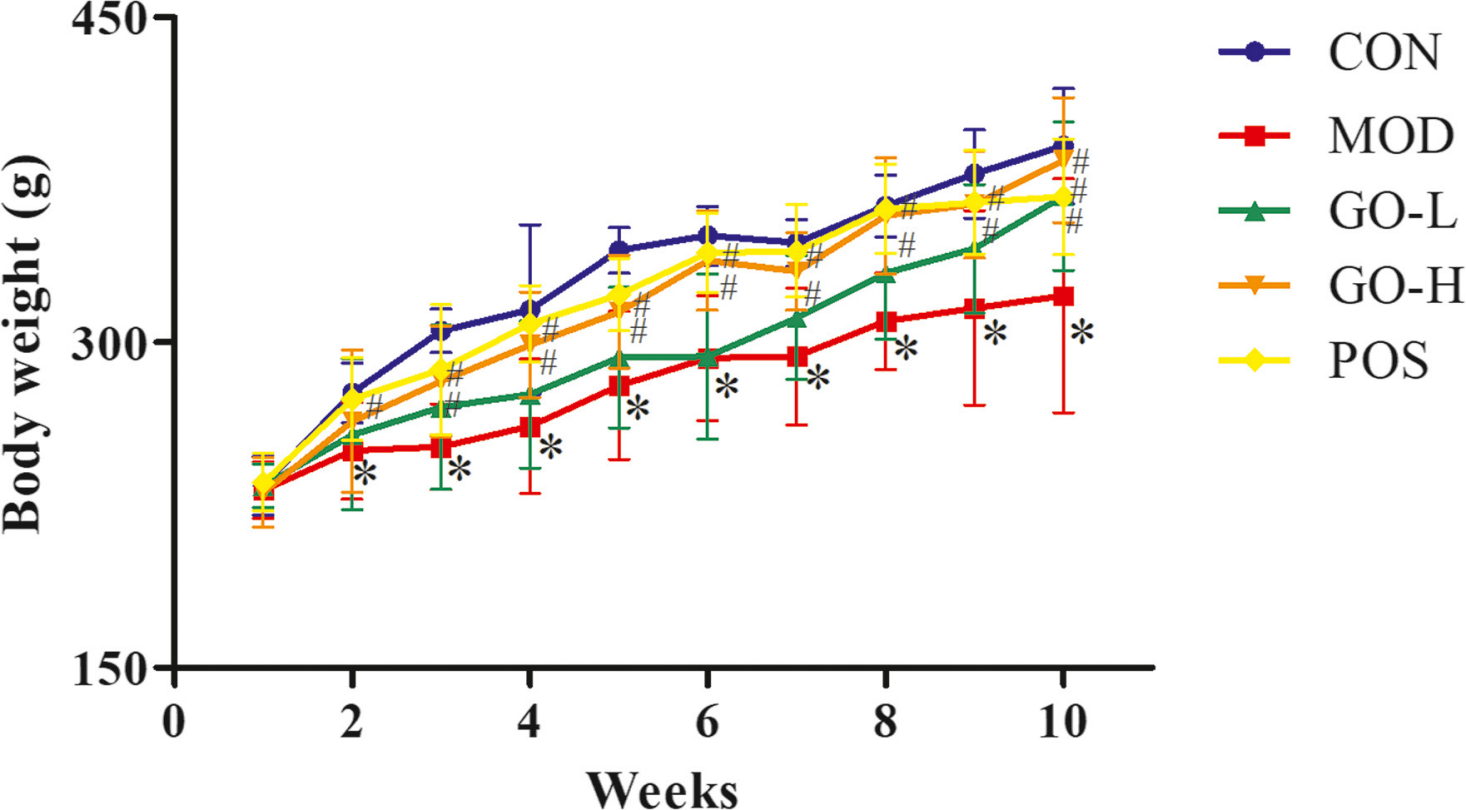
Effects of GO on body weight in rats exposed to alcohol. CON, control group; MOD, model group; GO‐L, low‐dose GO group; GO‐H, high‐dose GO group; POS, positive group. Values are expressed as means ± *SD*. ^*^ *p* < .05 compared with the CON group. ^#^ *p* < .05 compared with the MOD group. ^△^ *p* < .05 compared with the POS group

The mean weight change of rat in each group was shown in Figure 2. The mean body weight of the MOD group was generally lower than the CON group and other groups. The mean weight of the MOD group was significantly lower than the CON group from the second week (*p* < .05) and the weight of the GO‐H group was significantly higher than the MOD group from the third week (*p* < .05). The mean weight of the POS group was significantly higher than the MOD group from the second week (*p* < .05). There was no difference between the GO‐H and POS groups (*p* > .05).

### 3.2 Effects of GO on liver index

**FIGURE 3:**
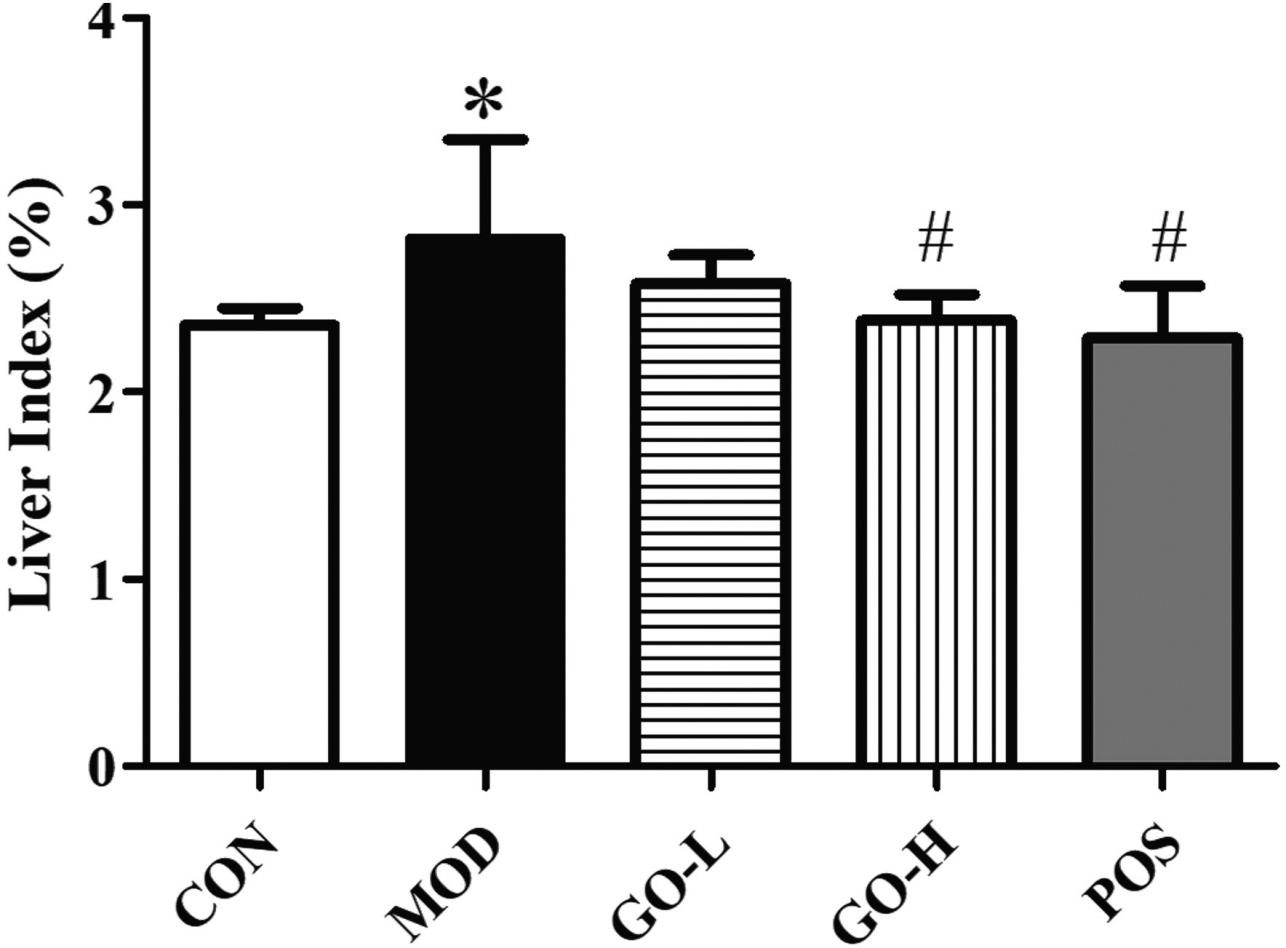
Effects of GO on liver index in rats exposed to alcohol. CON, control group; MOD, model group; GO‐L, low‐dose GO group; GO‐H, high‐dose GO group; POS, positive group. Values are expressed as means ± *SD*. ^*^ *p* < .05 compared with the CON group. ^#^ *p* < .05 compared with the MOD group. ^△^ *p* < .05 compared with the POS group

As shown in Figure 3, the liver index in the MOD group was significantly increased compared with the CON group (*p* < .05). The liver index was significantly reduced in the GO‐H and POS groups compared to the MOD group (all *p*s < .05). There was no difference between the GO‐H and POS groups and the GO‐H group and POS group were not different from the CON group (*p* > .05).

### 3.3 Effects of GO on liver function

As shown in Table 1, the levels of ALT and AST in the MOD group were significantly increased compared with the CON group (*p* < .05). Serum ALT and AST in the GO‐H and POS groups were significantly decreased compared with the MOD group (*p* < .05). The levels of GGT and CHE in the MOD group were significantly increased compared with the CON group (*p* < .05). The GGT and CHE in the GO‐H group decreased to a certain extent compared with the MOD group, but there was no statistical significance (*p* > .05). No significant difference was found in ALT, AST, and CHE between the GO‐H group and the POS group (*p* > .05).

**TABLE 1:** Effects of GO on liver function indicators in rats exposed to alcohol

Groups	ALT (U/L)	AST (U/L)	GGT (U/L)	CHE (U/L)
CON	35.38 ± 7.71	111.50 ± 26.43	2.69 ± 0.65	84.17 ± 9.53
MOD	61.67 ± 7.97*	153.17 ± 20.55*	4.12 ± 0.53*	123.33 ± 16.35*
GO‐L	59.00 ± 12.50^△^	127.30 ± 32.32	4.05 ± 0.68^*△^	118.60 ± 13.65^*△^
GO‐H	45.80 ± 7.21^#^	122.60 ± 24.72^#^	3.77 ± 0.85^*△^	109.67 ± 11.50*
POS	38.30 ± 9.60^#^	114.00 ± 17.44^#^	2.87 ± 0.87^#^	95.60 ± 10.97^#^

Note

Values are expressed as means ± *SD*.

Abbreviations: CON, control group; MOD, model group; GO‐L, low‐dose GO group; GO‐H, high‐dose GO group; POS, positive group. ALT, alanine aminotransferase; AST, glutamic oxalacetic transaminase; GGT, gamma‐glutamyl transferase; CHE, cholinesterase.

* *p* < .05 compared with the CON group.

^#^ *p* < .05 compared with the MOD group.

^△^ *p* < .05 compared with the POS group.

### 3.4 Effect of GO on serum lipid metabolism

As shown in Table 2, serum TG level in the MOD group was significantly increased compared with the CON group (*p* < .05). Serum TG levels were significantly reduced in the GO‐L and GO‐H groups compared with the MOD group (all *p*s < .05), but the serum TG levels of the GO‐L group and GO‐H group were not different from the CON group. There were no significant differences in TC, HDL‐C, and LDL‐C among the groups (*p* > .05).

**TABLE 2:** Effects of GO on serum lipid metabolism in rats exposed to alcohol

Groups	TG (mmol/L)	TC (mmol/L)	HDL‐C (mmol/L)	LDL‐C (mmol/L)
CON	0.32 ± 0.10	1.19 ± 0.25	0.59 ± 0.20	0.19 ± 0.03
MOD	0.54 ± 0.17*	1.29 ± 0.29	0.58 ± 0.16	0.22 ± 0.04
GO‐L	0.35 ± 0.10^#^	1.14 ± 0.13	0.51 ± 0.07	0.18 ± 0.03
GO‐H	0.29 ± 0.03^#△^	1.12 ± 0.20	0.50 ± 0.13	0.19 ± 0.05
POS	0.44 ± 0.02*	1.26 ± 0.20	0.52 ± 0.11	0.20 ± 0.03

Note

Values are expressed as means ± *SD*.

Abbreviations: CON, control group; MOD, model group; GO‐L, low‐dose GO group; GO‐H, high‐dose GO group; POS, positive group. TG, triacylglycerol; TC, total cholesterol; HDL‐C, high‐density lipoprotein cholesterol; LDL‐C, low‐density lipoprotein cholesterol.

* *p* < .05 compared with the CON group.

^#^ *p* < .05 compared with the MOD group.

^△^ *p* < .05 compared with the POS group.

### 3.5 Effects of GO on hepatic TG

**FIGURE 4:**
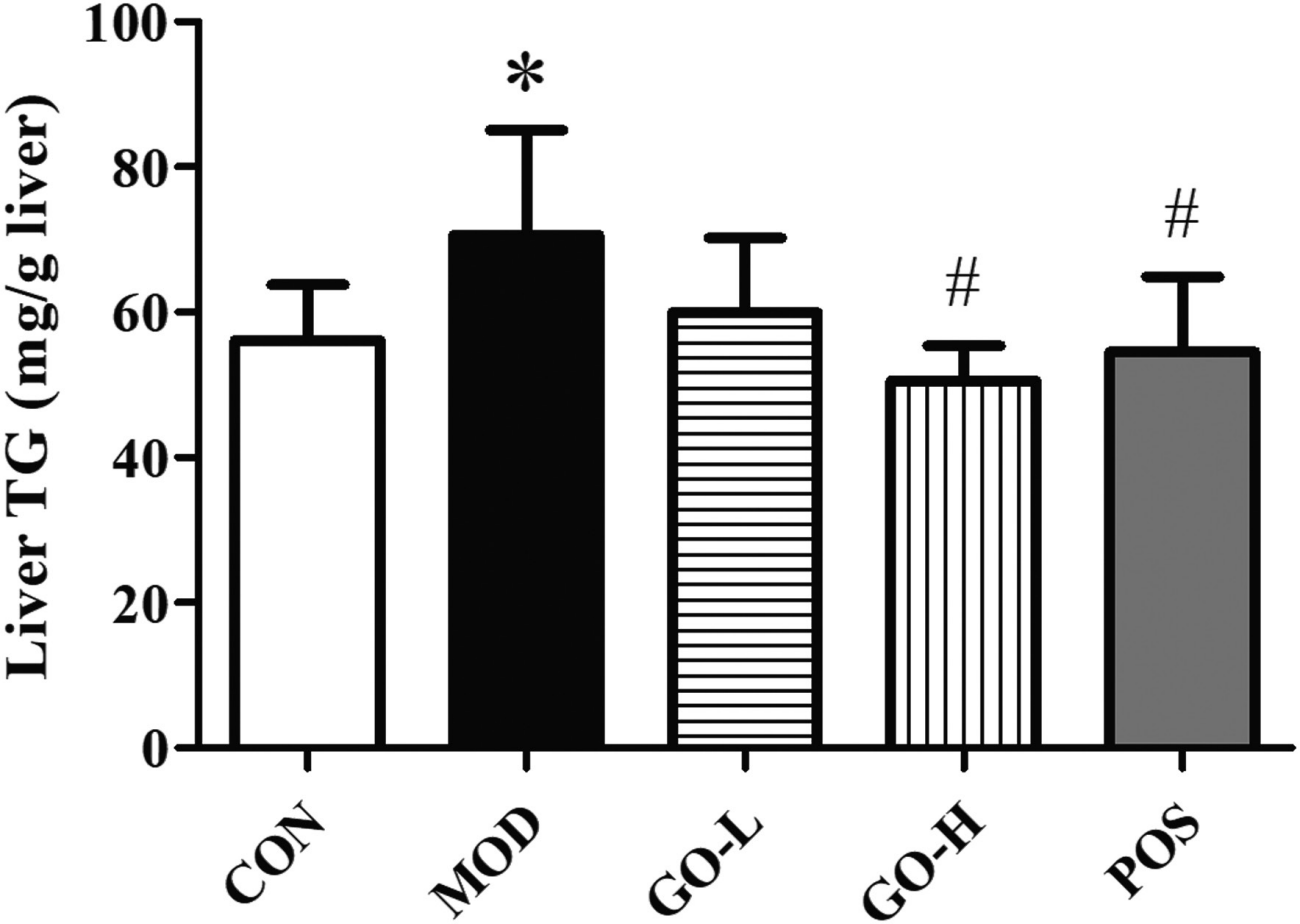
Effects of GO on hepatic TG in rats exposed to alcohol. CON, control group; MOD, model group; GO‐L, low‐dose GO group; GO‐H, high‐dose GO group; POS, positive group; TG, triacylglycerol. Values are expressed as means ± *SD*. ^*^ *p* < .05 compared with the CON group. ^#^ *p* < .05 compared with the MOD group. ^△^ *p* < .05 compared with the POS group

The TG level in the MOD was significantly increased compared with the CON group (*p* < .05) as shown in Figure 4. Hepatic TG was significantly reduced in the GO‐H and the POS groups compared with the MOD group (*p* < .05). There was no difference between the GO‐H and POS groups compared with the CON group, respectively (*p* > .05).

### 3.6 Effects of GO on serum oxidative stress index

As shown in Table 3, the level of serum MDA was significantly increased in the MOD group compared with the CON group (*p* < .05), but the serum MDA level in the POS and GO‐H groups were significantly reduced compared with the MOD group (*p* < .05). The level of serum GSH‐Px in the MOD group was significantly reduced compared with the CON group (*p* < .05), but the level of serum GSH‐Px in the GO‐H group was significantly increased compared with the MOD group (*p* < .05). The level of serum SOD was significantly reduced in the MOD group compared with the CON group (*p* < .05), but the level of serum SOD was significantly increased in the GO‐L, GO‐H, and POS groups compared with the MOD group (*p* < .05). The levels of serum MDA, GSH‐Px, and SOD in the GO‐H and POS groups were not different compared with the CON group (*p* > .05).

**TABLE 3:** Effects of GO on serum oxidative stress index in rats exposed to alcohol

Groups	MDA (nmol/ml)	GSH‐Px (U/ml)	SOD (U/ml)
CON	4.56 ± 0.29	1750.84 ± 275.58	360.21 ± 10.05
MOD	5.68 ± 0.71*	1243.00 ± 200.63*	331.55 ± 28.61*
GO‐L	5.17 ± 0.68	1196.54 ± 257.27*	365.29 ± 10.34^#^
GO‐H	4.52 ± 0.66^#^	1573.68 ± 164.04^#^	367.71 ± 18.02^#^
POS	4.78 ± 0.72^#^	1468.23 ± 281.58	373.95 ± 23.71^#^

Note

Values are expressed as means ± *SD*.

Abbreviations: CON, control group; MOD, model group; GO‐L, low‐dose GO group; GO‐H, high‐dose GO group; POS, positive group; MDA, malondialdehyde; GSH‐Px, glutathione peroxidase; SOD, superoxide dismutase.

* *p* < .05 compared with the CON group.

^#^ *p* < .05 compared with the MOD group.

^△^ *p* < .05 compared with the POS group.

### 3.7 Effects of GO on hepatic oxidative stress index

**FIGURE 5:**
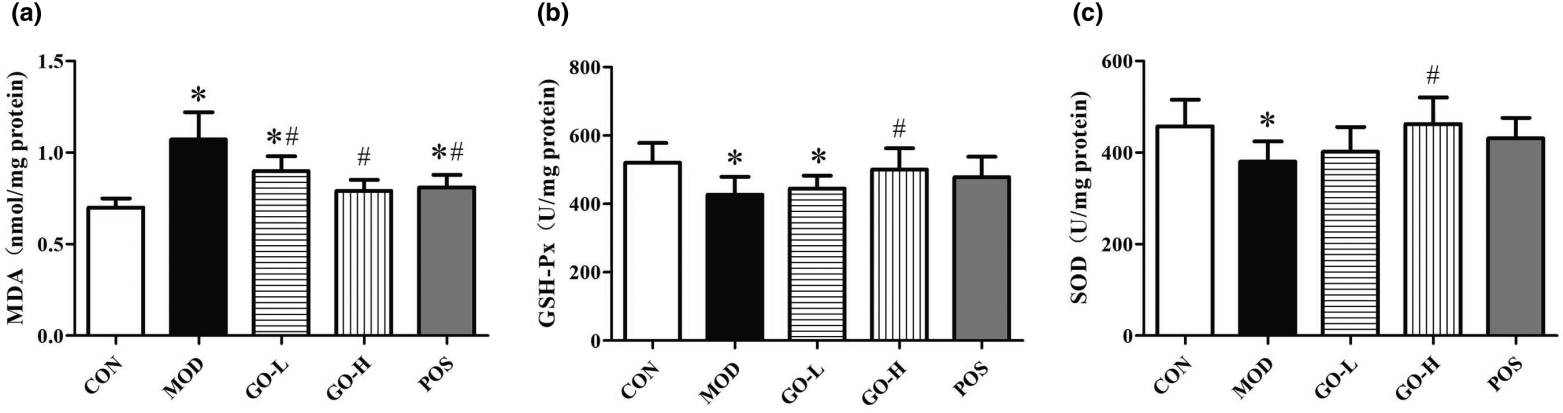
Effects of GO on hepatic oxidative stress index in rats exposed to alcohol. (a) Malonaldehyde (MDA) in liver, (b) glutathione peroxidase (GSH‐Px) in liver, (c) superoxide dismutase (SOD) in liver. CON, control group; MOD, model group; GO‐L, low‐dose GO group; GO‐H, high‐dose GO group; POS, positive group; MDA, malondialdehyde; GSH‐Px, glutathione peroxidase; SOD, superoxide dismutase. Values are expressed as means ± *SD*. ^*^ *p* < .05 compared with the CON group. ^#^ *p* < .05 compared with the MOD group. ^△^ *p* < .05 compared with the POS group

As shown in Figure 5, the level of MDA in the MOD group was significantly increased compared with the CON group (*p* < .05), but the levels of MDA in the GO‐L, GO‐H, and POS groups were significantly reduced compared with the MOD group (*p* < .05). The levels of GSH‐Px and SOD in the MOD group were significantly reduced compared with the CON group (all *p*s < .05). These alcohol‐induced effects were reversed by GO, because the levels of GSH‐Px and SOD in the liver of the GO‐H group increased compared with the MOD group (*p* < .05). The MDA, GSH‐Px, and SOD levels in the GO‐H group was not different from those in the CON group and the POS group (*p* > .05).

### 3.8 Effects of GO on the protein expression of Sirt1, PGC‐1α, and FoxO1

**FIGURE 6:**
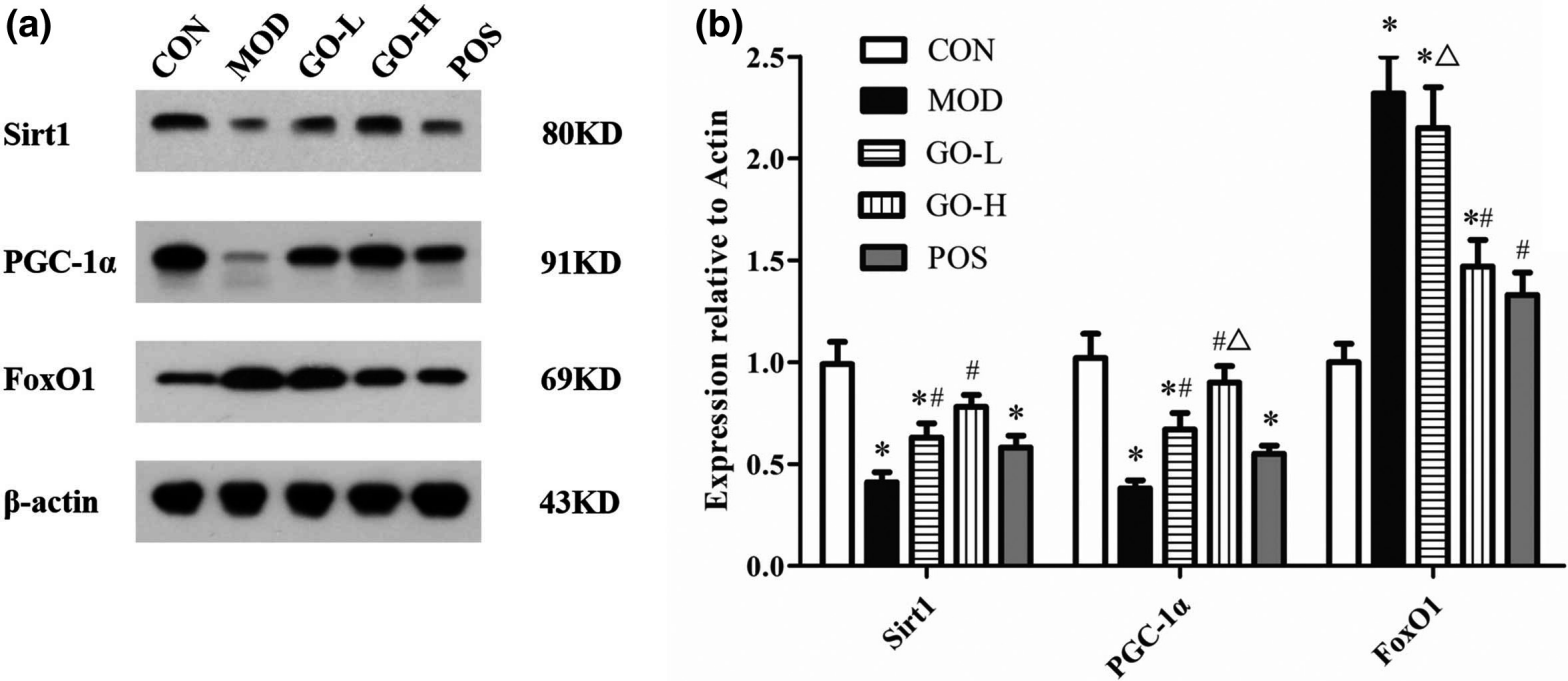
Effects of GO on Sirt1, PGC‐1α, and FoxO1 levels in rats exposed to alcohol. (a) Western blot analysis of Sirt1, PGC‐1α, and FoxO1 in liver tissue, (b) quantitative analysis of the western blot data. CON, control group; MOD, model group; GO‐L, low‐dose GO group; GO‐H, high‐dose GO group; POS, positive group; Sirt1, silent information regulator 1; PGC‐1α, proliferator‐activated receptor‐gamma coactivator‐1alpha; FoxO1, forkhead transcription factor O1. Values are expressed as means ± *SD*. ^*^ *p* < .05 compared with the CON group. ^#^ *p* < .05 compared with the MOD group. ^△^ *p* < .05 compared with the POS group

As shown in Figure 6, the levels of Sirt1 and PGC‐1α were significantly reduced in the MOD group compared with the CON group (*p* < .05). After supplementation of GO, the levels of Sirt1 and PGC‐1α in the GO‐H group were significantly increased (*p* < .05). There was no difference in Sirt1 and PGC‐1α levels in the GO‐H group compared with the CON group. The level of FoxO1 in the MOD group was significantly increased compared with the CON group (*p* < .05), but the levels of FoxO1 were significantly lower in the GO‐H and POS groups compared with the MOD group (*p* < .05). There was no difference in Sirt1 and FoxO1 levels between the GO‐H and POS groups (*p* > .05).

### 3.9 Effect of GO on oxidative stress index of colon tissue

**FIGURE 7:**
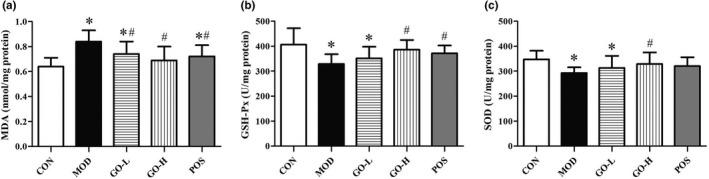
Effect of GO on oxidative stress index of colonic tissue in rats exposed to alcohol. (a) MDA, (b) GSH‐Px, and (c) SOD in colon tissue. CON, control group; MOD, model group; GO‐L, low‐dose GO group; GO‐H, high‐dose GO group; POS, positive group; MDA, malondialdehyde; GSH‐Px, glutathione peroxidase; SOD, superoxide dismutase. Values are expressed as means ± *SD*. ^*^ *p* < .05 compared with the CON group. ^#^ *p* < .05 compared with the MOD group. ^△^ *p* < .05 compared with the POS group

As shown in Figure 7, significantly increased MDA level and significantly decreased GSH‐Px and SOD were found in the MOD group compared with the CON group (*p* < .05). The GO‐H group had significantly decreased MDA level and significantly increased GSH‐Px and SOD levels compared with the MOD group (*p* < .05). The MDA, GSH‐Px, and SOD levels in the GO‐H group were not different from the CON and POS groups (*p* > .05).

### 3.10 Effects of GO on ZO‐1 and Claudin1 protein expression levels in colon tissues

**FIGURE 8:**
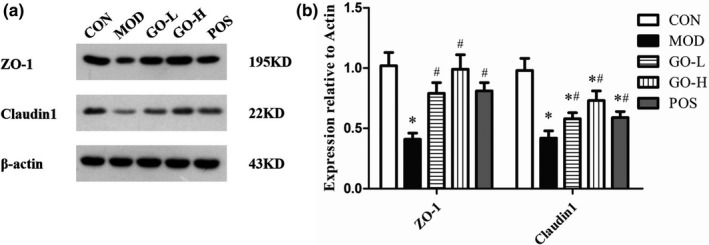
Effects of GO on ZO‐1 and Claudin1 levels in colon tissues of rats exposed to alcohol. (a) Western blot analysis of ZO‐1 and Claudin1 in colon tissue, (b) quantitative analysis of the western blot data. CON, control group; MOD, model group; GO‐L, low‐dose GO group; GO‐H, high‐dose GO group; POS, positive group; ZO‐1, Zonula occludens‐1. Values are expressed as means ± *SD*. ^*^ *p* < .05 compared with the CON group. ^#^ *p* < .05 compared with the MOD group. ^△^ *p* < .05 compared with the POS group

As shown in Figure 8, the expression level of ZO‐1 in the MOD group was significantly reduced compared with the CON group (*p* < .05). The expression levels of ZO‐1 were significantly higher in the GO‐L, GO‐H, and POS groups compared to the MOD group (*p* < .05) and there were no differences in the ZO‐1 level in the GO‐L, GO‐H, and POS groups compared with the CON group. Claudin1 expression levels were significantly reduced in the MOD, GO‐L, GO‐H, and POS groups compared with the CON group (*p* < .05). Claudin1 expression levels were significantly increased in the GO‐L, GO‐H, and POS groups compared with the MOD group (*p* < .05). There was no difference in ZO‐1 and Claudin1 expression levels between the GO‐H and POS groups (*p* > .05).

### 3.11 Effects of GO on α and β diversity of intestinal flora

**FIGURE 9:**
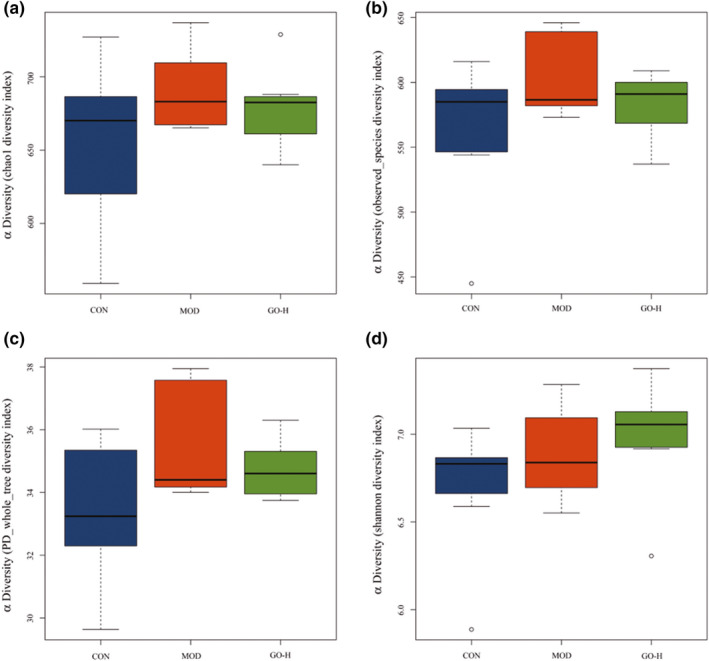
Effects of GO on α diversity analysis of intestinal flora in rats exposed to alcohol. (a) Chao1 index, (b) observed species index, (c) PD whole tree index, (d) Shannon index. CON, control group; MOD, model group; GO‐H, high‐dose GO group

The results of intestinal flora analysis of rats showed that the Good's coverage of the three groups based on OUT richness was greater than 99.50%, indicating that most taxa had been covered by the sample test. From the index analysis of chao1 (Figure 9a), observed species (Figure 9b), PD‐whole tree (Figure 9c), and Shannon (Figure 9d), there was no significant difference in the species richness and diversity between the MOD group and the GO‐H group. The results showed that GO did not significantly affect the structure of intestinal flora.

**FIGURE 10:**
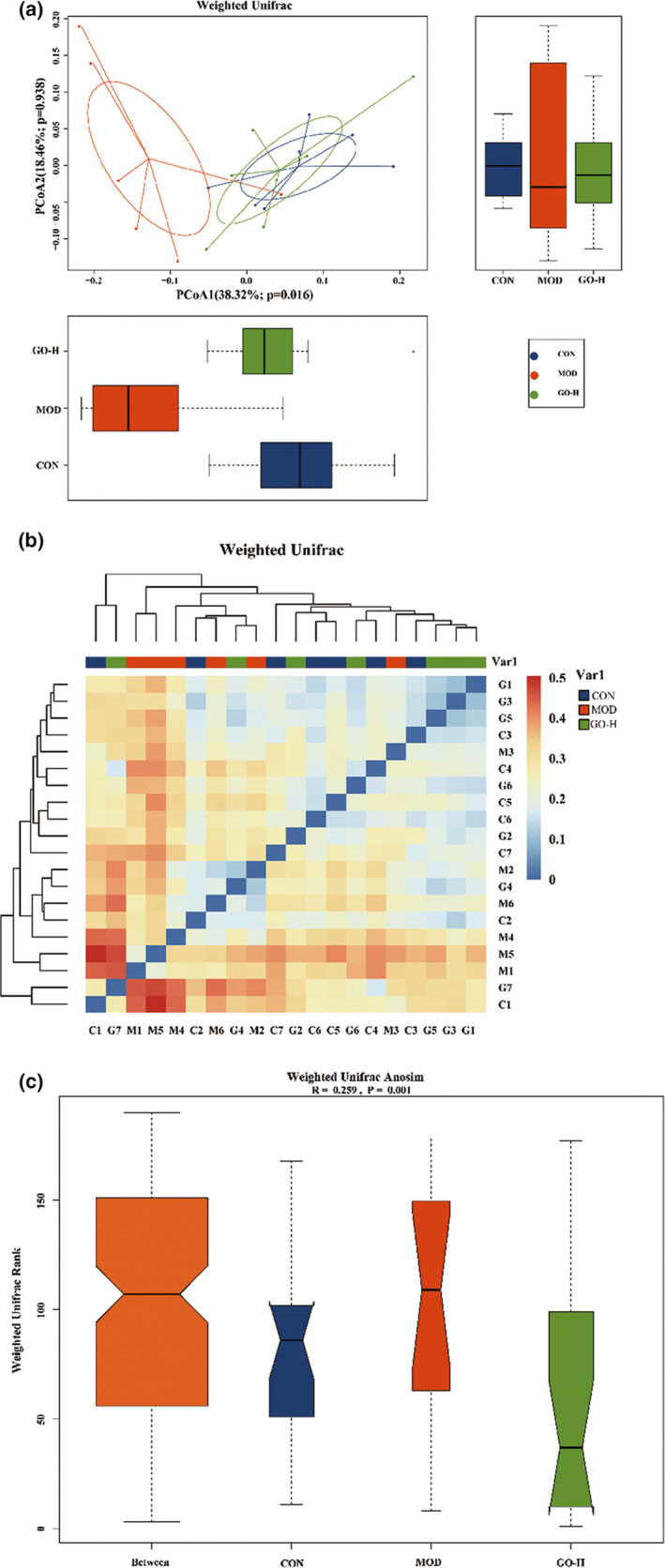
Effects of GO on β diversity analysis of intestinal flora in rats exposed to alcohol. (a) Principal coordinate analysis (PCoA) of intestinal flora. (b) Heat map of UniFrac distance based on 20 samples. (c) ANOSIM analysis of similarity. CON, control group; MOD, model group; GO‐H, high‐dose GO group. *r* > 0 indicates that the difference between groups is greater than the difference within groups. *p* < .05 indicated that the difference between the groups was statistically significant

The results were also confirmed by principal coordinate analysis (PCoA) based on UniFrac distance and the analysis based on PCoA1 with a contribution to population variance of 38.32%, (*p* = .016) and PCoA2 with a contribution to population variance of 18.46% (*p* = .938) found that all samples were divided into three different clusters, but the GO‐H group was like the CON group. The results showed that the MOD group showed an obvious trend of separation in spatial distribution compared with the CON group, indicating that the intake of alcohol significantly changed the composition of intestinal microbial community in rats. The distance between the MOD group and CON group samples was larger than the distance between GO‐H group and CON group samples, indicating that the bacterial community structure of the GO‐H group rats was closer to that of the CON group rats than the MOD group (Figure 10a). The results of the Beta diversity heat map showed that the community composition of the intestinal flora of the GO‐H group was closer to the CON group than the MOD group, which was consistent with the results of the principal coordinate analysis in Figure 10(b). The ANOSIM analysis revealed different bacterial community structures among the three groups (*r* = 0.259, *p* = .001) as shown in Figure 10(c). All the above analyses showed that there were significant differences in the composition and structure of intestinal flora among the three groups, the MOD group was significantly different from the two groups, and the composition of intestinal flora community in the GO‐H group was like the CON group.

### 3.12 Effects of GO on species distribution and abundance

**FIGURE 11:**
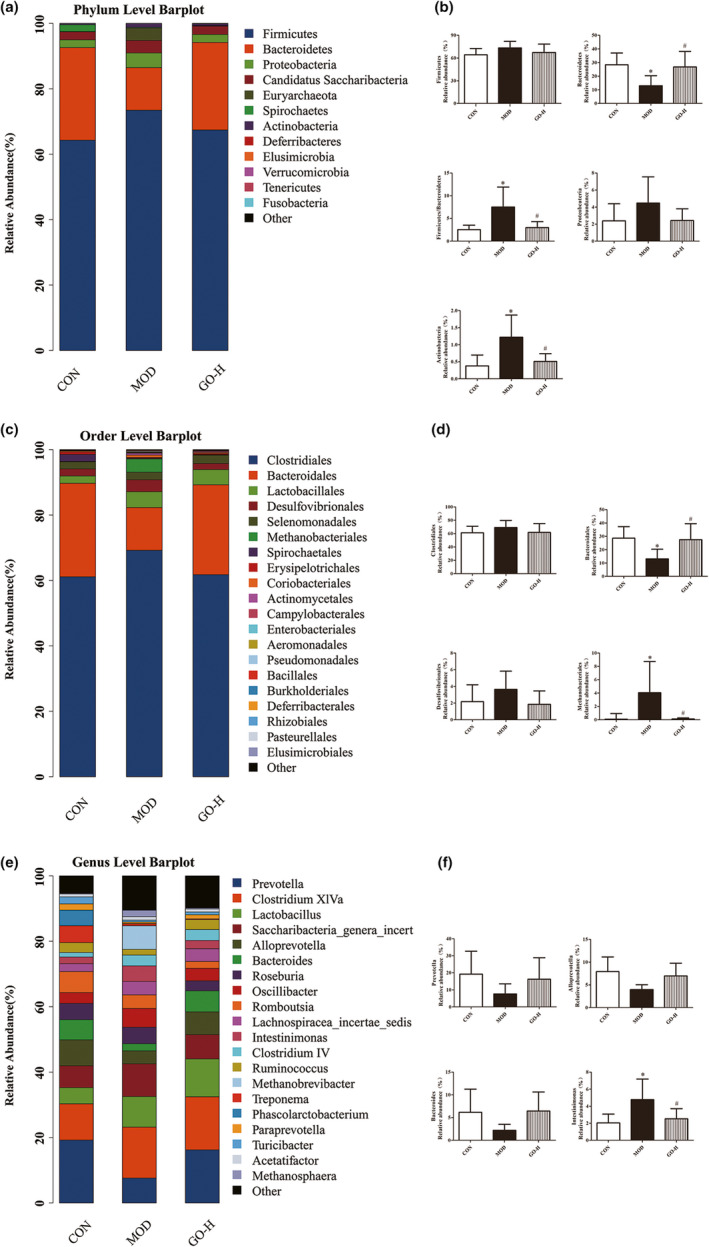
Effect of GO on species distribution and abundance at phylum, order, and genus levels of intestinal flora in rats exposed to alcohol. (a) Relative abundance of species at phylum level. (b) Analysis of variance histogram of relative abundance of species at phylum level. (c) Relative abundance of species at order level. (d) Analysis of variance histogram of relative abundance of species at order level. (e) Relative abundance of species at genus level. (f) Analysis of variance histogram of relative abundance of species at genus level. CON, control group; MOD, model group; GO‐H, high‐dose GO group. ^*^ *p* < .05 compared with the CON group. ^#^ *p* < .05 compared with the MOD group

As shown in Figure 11, at the phylum level, compared with the CON group, the abundance of *Firmicutes* in the MOD group relatively increased, while that in the GO‐H group had a slight decrease compared with the MOD group, but there was no statistical significance (*p* > .05). The level of *Bacteroidetes* in the MOD group was lower than that in the CON group and GO treatment increased the abundance of *Bacteroidetes* (*p* < .05). The ratio of *Firmicutes*/*Bacteroides* (F/B) in the MOD group was significantly higher compared to the CON group and that in the GO‐H group was significantly lower than that in the MOD group (*p* < .05). The *Actinobacteria* of the MOD group was significantly higher and the GO‐H group was significantly lower than the MOD group (*p* < .05) as shown in Figure 11(a,b).

At the order level, in comparison with the CON group, the *Clostridiales* in MOD group slightly increased, while that in the GO‐H group relatively decreased compared with the MOD group (*p* > .05), *Bacteroidales* in the MOD group reduced significantly, while *Bacteroidales* in the GO‐H group increased significantly compared with the MOD group (*p* < .05) and *Methanobacteriales* of the MOD group was significantly higher and the GO‐H group was significantly lower than the MOD group (*p* < .05) as seen in Figure 11(c,d).

At the genus level, compared with the CON and GO‐H groups, the MOD group microbiota was deficient in *Prevotella*, *Alloprevotella*, and *Bateroides* (*p* > .05). The *Intestinimonas* of the MOD group was significantly higher compared with the CON group, but the level of *Intestinimonas* was decreased compared to the MOD group (*p* < .05) as seen in Figure 11(e,f).

**FIGURE 12:**
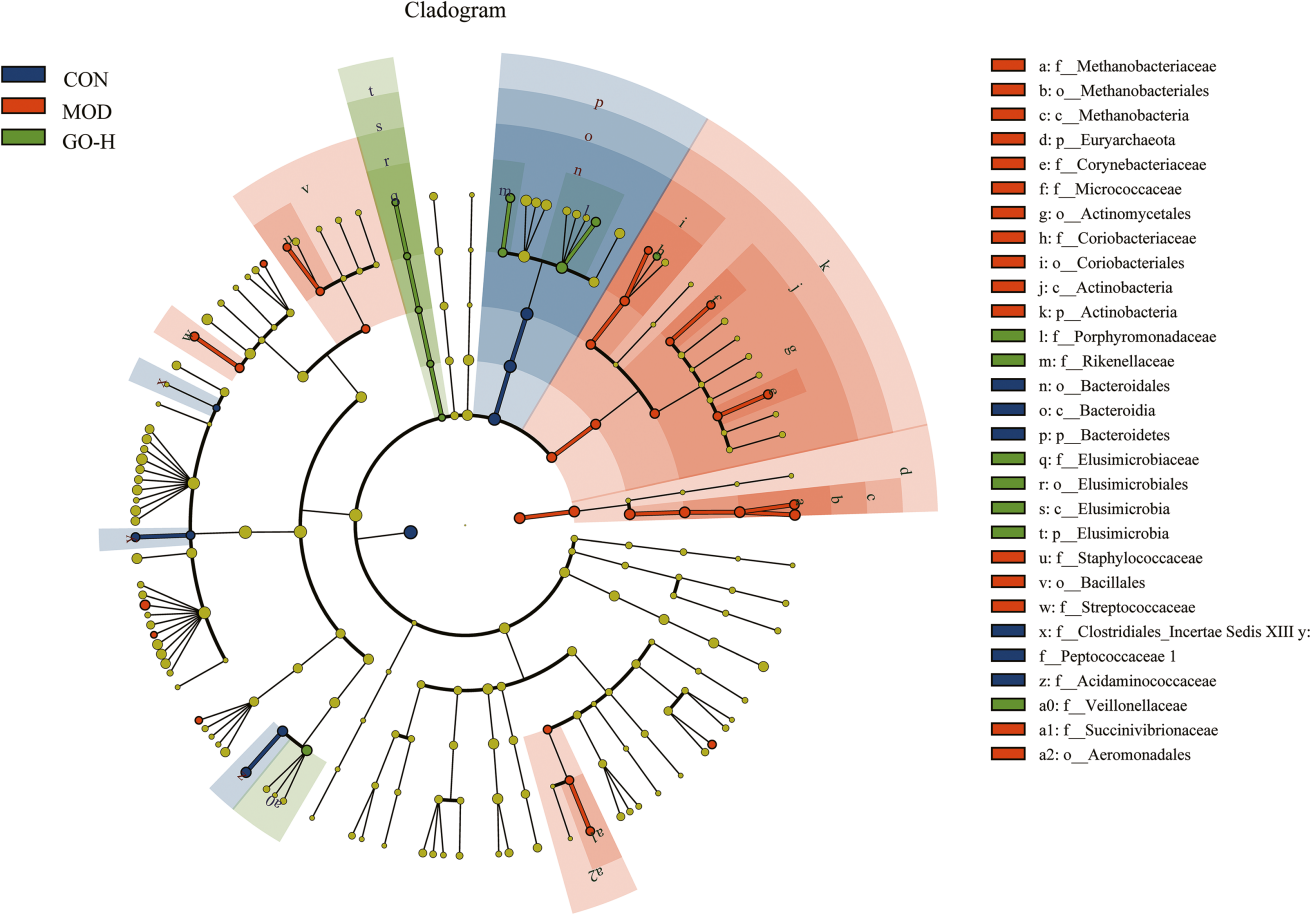
Effects of GO on the structure of intestinal flora in rats exposed to alcohol. CON, control group; MOD, model group; GO‐H, high‐dose GO group. The figure shows LEfSe sequence analysis. The biomarker groups are highlighted by colored circles and shaded areas

The LDA effect size (LEfSe) analysis showed that the enriched species in the CON group was *Bacteroidetes*, the enriched species in the MOD group included *Actinobacteria* and *Euryarchaeota* and the enriched species in the GO‐H group was *Elusimicrobia* (Figure 12).

### 3.13 Effect of GO on SCFAs

**FIGURE 13:**
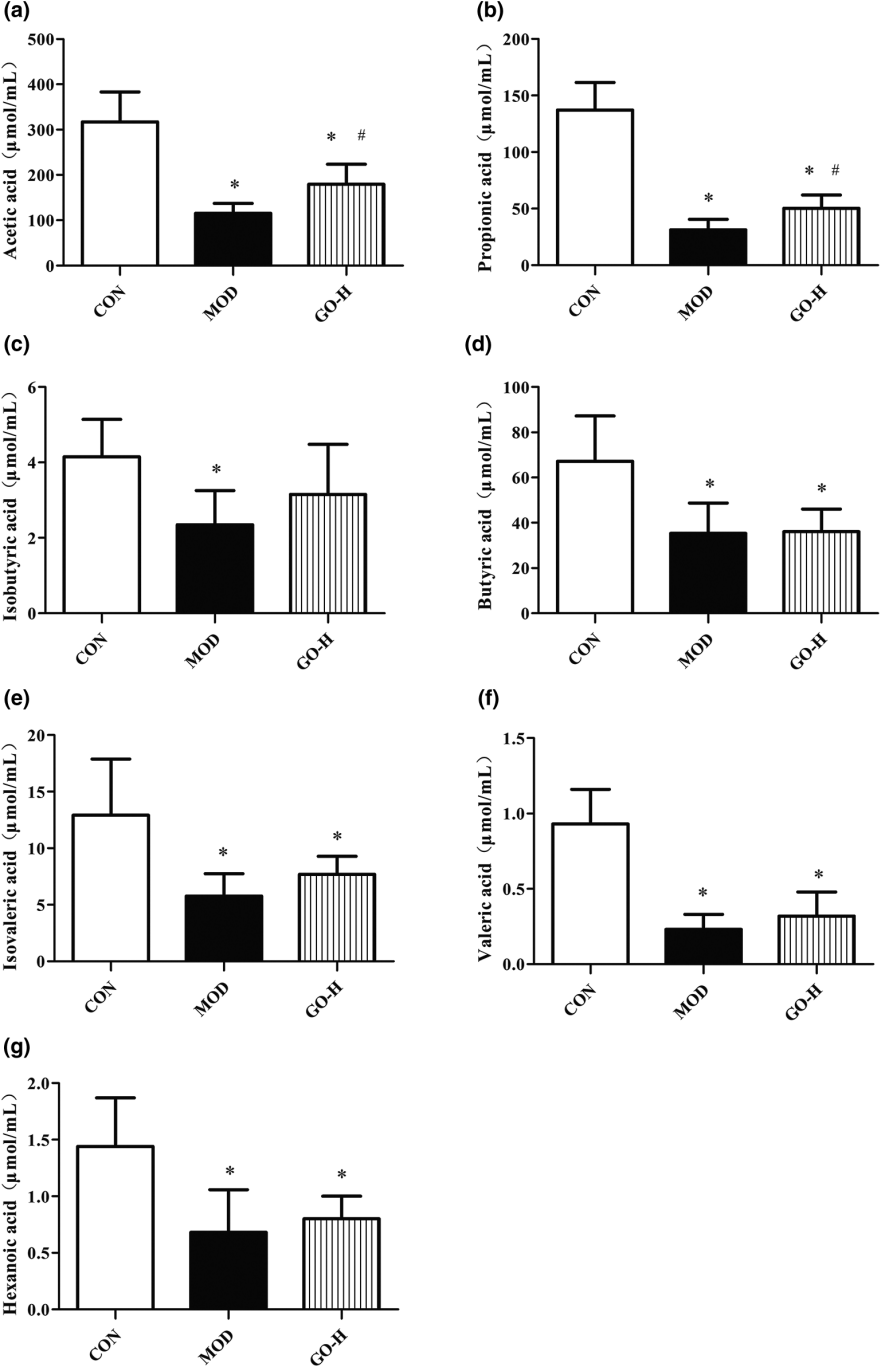
Effect of GO on the content of SCFAs in rats exposed to alcohol. (a) AA content in intestinal contents. (b) PA content in intestinal contents. (c) IBA content in intestinal contents. (d) BA content in intestinal contents. (e) IVA content in intestinal contents. (f) VA content in intestinal contents. (g) HA content in intestinal contents. CON, control group; MOD, model group; GO‐H, high‐dose GO group; AA, acetic acid; PA, propionic acid; IBA, isobutyric acid; BA, butyric acid; IVA, isovaleric acid; VA, valeric acid; HA, hexanoic acid. Values are expressed as means ± *SD*. ^*^ *p* < .05 compared with the CON group. ^#^ *p* < .05 compared with the MOD group

The levels of SCFAs including acetic acid (AA), propionic acid (PA), isobutyric acid (IBA), butyric acid (BA), isovaleric acid (IVA), valeric acid (VA), and hexanoic acid (HA) were analyzed. The results are shown in Figure 13. AA was the most abundant SCFAs, followed by PA and BA. The contents of SCFAs in the MOD group were all significantly decreased compared with the CON group (*p* < .05). The content of AA and PA in GO‐H group were significantly higher than that in the MOD group (*p* < .05) and other SCFAs were slightly higher than the MOD group (*p* > .05). Overall, SCFAs in the MOD group decreased significantly compared with the CON group and GO treatment could alleviate this trend.

## 4 DISCUSSION

In this study, GO alleviated high TG levels in blood and liver of alcohol‐exposed rats, because it ameliorated the oxidative stress by attenuating the alcohol‐induced Sirt1 inhibition. Furthermore, we found that GO could regulate intestinal barrier and intestinal flora, which might further improve high TG level in alcohol‐exposed rats.

Liver is the main organ responsible for the metabolism of ethanol. Ethanol and its metabolites such as acetaldehyde acetate and fatty acid ethanol ester are considered to have direct toxic effects on the liver (Rocco et al., 2014). Alcoholic liver injury will lead to the imbalance of liver lipid synthesis and decomposition, resulting in the disorder of lipid metabolism (Lieber, 2003; Liu et al., 2020). ALT and AST are the most sensitive indexes to reflect the injury of hepatocytes. GGT is usually used as a marker of whether patients can return to normal. In the convalescence stage of liver disease, GGT may still elevate abnormally even if ALT and AST return to normal levels (Zhao et al., 2021). Consistent with other studies (Kim et al., 2019), significantly high serum ALT, AST, GGT, CHE, TG levels, and the markedly elevated liver index and liver TG levels were found in alcohol‐exposed rats in this study, indicating that alcohol exposure caused liver injury and TG metabolism disorder in rats.

Garlic has been considered as a natural substance to resist various physiological threats since ancient times. A series of biological benefits of garlic have been reported, such as antitumor, antibacterial, and hypoglycemic activities (Ko et al., 2018). GO is produced by steam distillation of garlic and plays a role in regulating lipid metabolism. Intragastric administration of GO for 60 days could significantly reduce the serum TG level of male Sprague Dawley rats fed with high‐fat diet (Yang et al., 2018). In this study, the GO‐H group had significantly lower serum ALT, AST, TG levels, and markedly reduced liver index and liver TG levels compared with the MOD group, suggesting that high‐dose GO can improve liver injury and TG metabolism disorder caused by alcohol exposure in rats. A similar effect was found in the GO‐L group, but not as significant as the GO‐H group.

Lipid metabolism disorder is closely related to oxidative stress (Wan et al., 2021; Xue et al., 2020). Alcohol metabolism leads to the accumulation of reactive oxygen species, which gradually reduces the protection of cells against reactive oxygen species. When reactive oxygen species are produced to the extent that antioxidant enzymes cannot be eliminated, oxidative stress will occur (Tao et al., 2021). The physiological functions of GO have been widely studied, mainly due to its antioxidant activity (Agarwal et al., 2007; Park et al., 2005). In this study, we detected the levels of lipid oxidation end products (MDA) and antioxidant enzymes (GSH‐Px and SOD) in serum and liver tissue, and found that high‐dose GO could significantly reduce liver oxidative stress caused by alcohol exposure. Similar results have been found in some other studies. For example, GO could significantly reduce the high MDA level induced by alcohol in vivo and in vitro (Zeng et al., 2012). DATS at 10 μmol/kg or DADS at 100 μmol/kg could markedly increase the GSH‐Px levels in liver of acute liver injury model rats induced by carbon tetrachloride (Fukao et al., 2004). Therefore, it can be speculated that inhibition of oxidative stress may be one of the possible mechanisms by which GO acts against alcohol‐induced high TG levels.

Sirt1 is an NAD^+^‐dependent protein deacetylase and plays an important role in regulating liver lipid metabolism (Rada et al., 2018; Sathyanarayan et al., 2017). As a key metabolic sensor, Sirt1 directly combines cell metabolism with the transcriptional activity and of several key transcription factors and transcriptional coactivators involved in metabolic homeostasis (Ding et al., 2017). FoxO1, one of the forkhead transcription factors in the o‐box subfamily, regulates its own transcriptional activity through Sirt1‐mediated deacetylation to control antioxidant stress (Xu et al., 2020). The results of an in vitro trial suggested that Sirt1/FoxO1 may be one of the potential therapeutic targets for LPS‐induced oxidative stress injury in rat insulinoma cells (Mo et al., 2019). The Sirt1/FoxO1 pathway increases the levels of antioxidant enzymes SOD and GSH‐Px and reduces the level of MDA reducing the oxidative stress injury of H9C2 cardiomyocytes (Jiang et al., 2021). PGC‐1α is a transcription coactivator that can regulate the stability of oxidants and antioxidants by increasing the expression of superoxide dismutase‐2 and GSH‐Px (Liang et al., 2020; Tian et al., 2019). A study of a high‐glucose‐induced diabetic mouse model showed that resveratrol ameliorates podocyte damage via Sirt1/PGC‐1α‐mediated attenuation of mitochondrial oxidative stress (Zhang, Chi, et al., 2019). Activation of the Sirt1/PGC‐1α pathway induced by specific activator SRT 1720 reduced oxidative damage in intestinal epithelial cells and prevented oxidative stress‐mediated ROS production caused by H_2_O_2_ exposure (Liang et al., 2020).

In this study, GO significantly changed the decrease of Sirt1 and PGC‐1α proteins expression and the increase of FoxO1 protein expression induced by alcohol exposure, which suggested that GO could ameliorate oxidative stress through Sirt1/PGC‐1α and Sirt1/FoxO1 pathways. There was no significant difference in Sirt1 and PGC‐1α proteins expression between the POS and MOD groups, indicating that diammonium glycyrrhizinate had weak regulatory effect on Sirt1/PGC‐1α pathway. This is also the reason why there was a significant difference in PGC‐1α expression level between the GO‐H and POS groups. Sirt1 expression level was not markedly elevated, but FoxO1 was significantly reduced in the POS group compared with the MOD group, which suggested that diammonium glycyrrhizinate might regulate FoxO1 expression by some other upstream proteins, but not Sirt1. For example, salvianolic acid B could reduce oxidative stress response by regulating Sirt3/FoxO1 signaling pathway and play a role in the treatment of nonalcoholic steatohepatitis (Wang et al., 2017). Diammonium glycyrrhizinate has obvious hepatoprotective effect, which is widely used as a positive drug to alleviate liver injury. It is not the best choice to use diammonium glycyrrhizinate as a positive drug in terms of lipid metabolism disorder induced by alcohol exposure. There are limitations when explore the mechanism of regulating lipid metabolism. However, in this study, the comparison between the MOD and GO‐L/GO‐H groups can reflect the regulatory effect and mechanism of GO on TG levels to a certain extent. In future study, more appropriate positive drug can be chosen and more in‐depth research can be conducted.

Alcohol is also metabolized in the intestine, and can cause intestinal oxidative stress (Cho et al., 2021). The stimulation of long‐term oxidative stress destroys the living environment of the flora and eventually leads to intestinal barrier dysfunction (Zhuang et al., 2019). Endotoxin is transferred from the intestinal cavity to the liver through the portal vein, which leads to lipid metabolism disorder (Neyrinck et al., 2017). The ZO‐1 and claudin‐1 proteins are important proteins in tight junction proteins, which are responsible for maintaining cell morphology and maintaining tight junction structural integrity and can be used as indicators to evaluate intestinal barrier. In vitro experiments confirmed that the decrease of oxidative stress upregulates the expression of ZO‐1 protein (Wu et al., 2018) and in vivo experiments confirmed that the decrease of oxidative stress and the improvement of intestinal mucosal barrier function occurred simultaneously (Zhang, Chi, et al., 2019). In this study, we examined the levels of MDA and antioxidant enzymes and ZO‐1 and Claudin‐1 proteins expression levels in colon. The results suggest that GO can significantly alleviate intestinal oxidative stress induced by alcohol exposure and further markedly improve the intestinal barrier dysfunction.

Due to the occurrence of intestinal oxidative stress induced by alcohol exposure, intestinal epithelial cells passively diffuse oxidation products and stimulate the growth of aerobic bacteria, resulting in the imbalance of the proportion of the main intestinal flora and the increase of pathogenic bacteria. A large number of studies have shown that there is a correlation between intestinal flora and blood lipid levels (Wang et al., 2016). GO is metabolized in intestinal tract and can regulate intestinal flora (Hsu et al., 2021). In this study, intestinal flora sequencing analysis was performed on the CON, MOD, and GO‐H groups. The results showed that the composition of intestinal flora in the MOD group was significantly different from the CON group. Similar results can be found in a population‐based study, which showed that in patients with alcoholic fatty liver disease, the species and quantity of *Bacteroides* and *Prevotella* decreased significantly, but the species and quantity of *Clostridiales* and *Proteobacteria* increased significantly (Chen et al., 2011). In this study, we found that high‐dose GO can significantly increase the level of *Bacteroides*, *Prevotella*, and *Alloprevotella* and reduce the level of *Clostridiales* and *Proteobacteria*. *Alloprevotella* was negatively correlated with blood and liver lipids and *Clostridiales* was positively correlated with liver lipids (Neyrinck et al., 2017). *Firmicutes* and *Bacteroides* are the main microflora in human intestinal flora and the abundance of *Firmicutes* was related to lipid accumulation and TG level (Jiang, Chen, et al., 2020). An increase of F/B ratio has been found in patients with nonalcoholic fatty liver disease. In this study, high‐dose GO can significantly reduce the increase of *Firmicutes* and F/B ratio induced by alcohol exposure. The results of intestinal flora sequencing analysis showed that GO can effectively regulate intestinal flora associated with lipid metabolism, which may be related to the alleviation of alcohol‐induced high TG levels. The results of LEfSe analysis found that the species of *Euryarchueota* and *Actinobacteria* were the two most abundant bacteria in the MOD group and whether they were potential markers of lipid metabolism disorders caused by alcohol exposure remains to be further studied.

SCFAs produced by intestinal bacteria including predominantly BA, followed by PA and AA can activate G protein‐coupled receptors, activate regulatory T cells and continuously strengthen the mucosal barrier, alleviating lipid metabolism disorders (Kelly et al., 2015). Intestinal flora promotes liver fatty acid metabolism by providing high levels of AA as a precursor for the synthesis of palmitate and stearate (Kindt et al., 2018). A study of C57BL/6J mice found that SCFAs inhibited lipogenesis by activating hepatic cAMP and improved oxidative stress, thereby improving lipid levels (den Besten et al., 2015). The reduction of intestinal flora such as *Bacteroides*, *Ruminococcus*, and *Prevotella* will affect the level of SCFAs (Morrison & Preston, 2016). In this study, the levels of AA, PA, IBA, BA, IVA, VA, and HA were significantly decreased in the MOD group, which may be related to the decreased bacterial abundance of bacteria such as *Bacteroides*, *Ruminococcus*, and *Prevotella* in the MOD group. GO significantly increased AA and PA production and the contents of IBA and IVA also increased to a certain extent by changing the abundance of *Bacteroides*, *Ruminococcus*, and *Prevotella*. GO regulates the intestinal flora and its products SCFAs, which may contribute to alleviate TG metabolism disorders.

### 4.1 Conclusions

In summary, GO can ameliorate liver oxidative stress by regulating Sirt1 and its downstream proteins FoxO1 and PGC‐1α, and further alleviate the high TG levels in alcohol‐exposed rats. GO can repair the mechanical barrier of intestinal mucosa and regulate intestinal flora, which may be related to the alleviation of high TG levels in alcohol‐exposed rats.

## CONFLICT OF INTEREST

The authors declare no conflicts of interest.

## ETHICAL REVIEW

This study was approved by the Animal Care and Use Committee of the Medical College of Qingdao University.
